# Distributed generation adverse impact on the distribution networks protection and its mitigation

**DOI:** 10.1016/j.heliyon.2022.e09624

**Published:** 2022-05-31

**Authors:** Haymanot Takele

**Affiliations:** School of Electrical and Computer Engineering, Debre Markos University, Debre Markos, Ethiopia

**Keywords:** Distributed generation, Protection of distribution network, Reverse power relay, Unidirectional fault current limiter

## Abstract

Integration of Distributed Generation (DG) generates problems for the protection of Distribution Networks (DNs) in power systems. When DG is integrated into a power distribution system (DS), the radial nature of the network is altered and the power starts to flow in a reverse direction. In addition to the reverse flow of power, the DG upsurges the fault current level and affects the existing time intermission coordination of the protective overcurrent relay. This study proposes a reverse power relay (RPR) and Fault Current Limiter (FCL) to mitigate the fault current level and reverse power flow in a Distribution Network (DN) by the use of DG. The FCL works only as a unidirectional fault current limiter (UFCL) by restricting the flow of fault current that occurs in the main grid (MG) of the DG. However, FCLs can protect the flow of fault currents from the MG by affecting the operational flexibility and reliability of the DG. To overcome the flow of fault current of the DG, this study proposes RPR to monitor the power flow to the DG. Collectively the study focuses on the protection of radial DS by using RPR and UFCL. The analysis and the modeling were conducted on the 15KV DN of the radial feeder in Debre Markos DN. The 3Φ fault analysis (which is more severe than others) was performed to validate the protection capability of the mitigation techniques which were proposed in this study.

## 1 Introduction

As stated by IEEE, DG is used when a generation of electricity is less than the capacity of central generation which can be improved by connecting nearby power systems of the distribution. DG can be classified based on their mode of operation (grid-connected and standalone mode), coupling type (directly and inverter joined), terminal characteristics (inject real power (P) only, inject reactive power (Q) only, inject both P & Q and consuming Q and injecting P)) and power ratings (includes DG power rating which has a scale of greater than 50MW which is allocated as large type and a DG power rating with a magnitude of less than 5KW which is assigned as micro type). Integration of DG in a radial distribution system has both positive and negative impacts on the power system. One of the adverse impacts of DG is losing the radial nature of power flow and increasing the level of fault current. The addition or integration of DG to the DN causes an increase in the level of fault current, and reliability drop, and affects the security of the protection system. The level of failure of equipment is dependent on the type, size, location, and number of DG in a power system. Fault currents can cause great damage to electrical equipment without the use of protective devices. Traditional DN does not consider the futurity of the protection system due to its costly nature. To integrate DG considering the protection system and its overload ratings, this study incorporates the use of UFCL and RPR. When a fault occurs in a DS, FCL limits the short circuit current effectively, but in a DG, current limitation by FCL during faults may degrade the reliability and flexibility of the system. For this reason, we propose the use of FCL as UFCL for the effective implementation of DG. This will help improve the reliability and flexibility of DN by eliminating the possibility of faults from getting to the main grid. Although the UF is useful in DN, it is disabled when the system encounters heavy loading. This drawback of the UFCL is alleviated by using RPR. The RPR limits the fault current during a fault in the DG. The DN by nature is unidirectional and the power flows from the generation station to the customer or power demand. The worst scenario occurs when a fault occurs and the total DG power output is greater than the DG load. This causes the protection system to be unable to detect faults protect the system. This study attempts to solve the above challenges by mitigating the adverse impact of DG on the protection of DN using UFCL and RPR. For analysis and experimentation, Debre Markos (D/M) DN which has a single 15KV radial feeder was taken as the case study. We only considered the solar and wind type of DG. These DGs were placed at selected weak buses which were selected using the loss sensitivity factor. The mitigation techniques were applied to the optimally sized and located DGs using particle swarm optimization. Only the fault level increment caused by the integration of DG is considered. The fault current level analysis was performed in the presence and absence of DG on the base case. This research work focuses on feeder 4 which carries 3.3MW (3.88MVA) and has 62 buses with it. The number of DGs is two (wind and solar). The DGs connected at the weakest bus which is bus 62.

## 2 Literature review

Zayandehroodi et al. [1] reviewed the impact of DG on the performance of strength gadget protection (PSP). The excessive wide variety of DGs was once found to damage the operation and protection of energy systems. This effect used to be considered to motivate fault current increment and trade the load glide direction. Furthermore, the addition of DG in the strength network led to hostilities between the DG and scheme of safety owing to an unexpected increment of brief circuit currents, protection machine coordination deficiency, and line reclosing ineffectiveness after a fault anundesired islanding generator protection interface tripping. The integration of DG into the DN resulted in the mal-operation giving a sudden float of fault modern-day which was no longer discovered in the time of designing the original or regular DN. In addition to the other parameters and machine prior voltage earlier than the fault, the increment of fault contemporary additionally relies upon a number of elements such as dissemination, capacity, technology, interface, and factor of connection of the DG.

Li et al. [2] carried out a learn about the challenge of energy flow direction, the mode of operation, and device fault traits as a result of DG integration leading to low voltage DN.

Khatod and Sharma [3] reviewed the high-quality and detrimental impacts of DG on DN. They learn published that the detrimental consequences of integrating DG are uneven coordination of protection and troubles with dynamic balance and islanding. Theirs learn about concluded that integrating DG into the current DN needs to consider the composite influences (i.e. technical, economic, and environmental).

Bak-Jensen et al. [4] studied the lack of coordination of safety gadgets coping with and finding out the high-quality protection scheme for the DN with DGs. We investigated the overall performance of protective devices and distinctive DG units with the aid of enforcing a model developed the usage of the DIgSILENT strength issue simulation tool.

Abdel-Salam et al. [5] proposed UFCL for the improvement of safety coordination in the presence of DG to shield the incidence of fault caused with the aid of the current increment. The paper used a method for finding the foremost resistance fee of UFCL with proper coordination of time intervals and also used both MATLAB and ETAP for simulation and analysis.

Zehir et al. [6] introduced a microgrid management system for the unfavorable effect mitigation of DG on DN for low voltage levels. The paper discussed how DG with low voltage ranges motives voltage restriction violation, community loss increment, and line overloads.

Sudhakar, et al. [7] mentioned the operation of RPR for the reduction of the impact of DG on DN and answered some technical, financial, and regulatory questions. To reduce the eft on DG, we proposed RPR as a safety mechanism to protect against voltage fluctuation and reversal strength conditions. Also, RPR was once used to decrease the effect of DG on DN protection by using introducing three management techniques, namely: pulse width modulation generator, distinct PI controller, and 3Φ region order analyzer for fault network sensing.

## 3 Modelling the test system and data analysis of feeder 4

**Figure 1:**
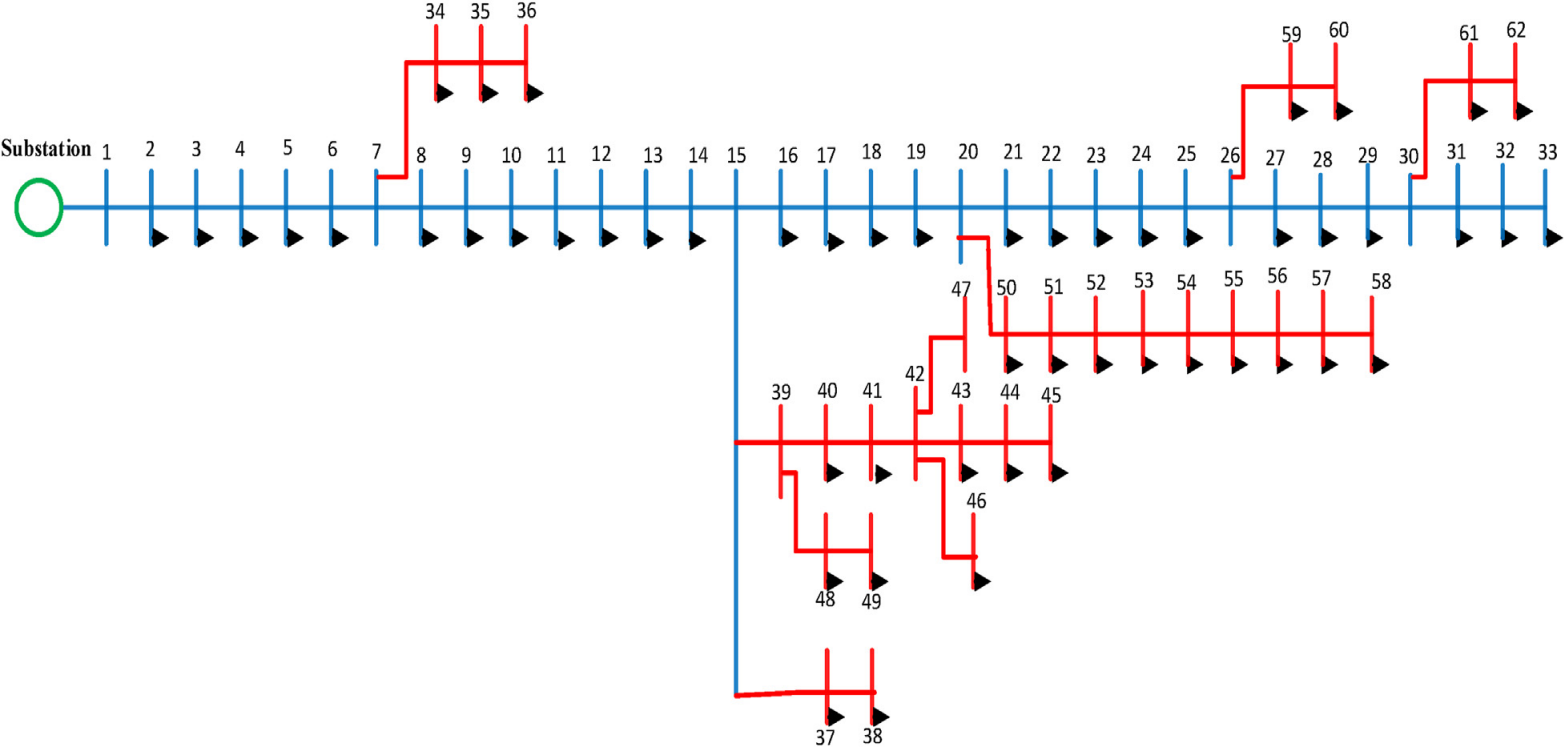
62 bus test system of the research work.

This paper considers feeder four of the Debre Markos distribution network which consists of 62 buses where the single line diagram of the feeder is shown in Figure 1. The distribution network in the feeder is configured radially. All buses are rated with 15KV and a step-down transformer is connected to it to step down and supply to the customers.

### 3.1 Fault analysis of distribution network with integrations of DG

DN is one of the main parts of the electric power system which is characterized by the design of short circuit capacity with the relation of thermal and mechanical equipment competence and constructions. When the grid contribution is minimum to the short circuit, reversely the DG contribution to the short circuit is maximum [6]. To compensate and simplify various assumptions of the methodology, IEC 60909 introduces the factor of correction for which multiply the respective impedances in all calculation formulae. These are synchronous generators (KG), transformers (KT), and power station units (KS and KSO).

(1) $${\text{K}}_{\text{T}}=0.95\text{∗}\frac{{\text{c}}_{\text{max}}}{1+0.6{\text{x}}_{\text{T}}}=\frac{{\text{U}}_{\text{n}}}{{\text{U}}_{\text{b}}}\text{∗}\frac{{\text{c}}_{\text{max}}}{1+{\text{x}}_{\text{T}}\left(\frac{{\text{I}}_{\text{T}}^{\text{b}}}{{\text{I}}_{\text{nT}}}\right)\sin\;{\text{φ}}_{\text{T}}^{\text{b}}}$$

(2) $${\text{K}}_{\text{G}}=\frac{{\text{U}}_{\text{n}}}{{\text{U}}_{\text{nG}}}\text{∗}\frac{{\text{c}}_{\text{max}}}{1+{\text{x}}_{\text{d}}^{\text{″}}\;\sin{\text{φ}}_{\text{nG}}}$$

(3) $${\text{K}}_{\text{S}}=\frac{{\text{U}}_{\text{nQ}}^{2}}{{\text{U}}_{\text{nG}}^{2}}\text{∗}\frac{1}{{\text{m}}_{\text{T}}^{2}}\text{∗}\frac{{\text{c}}_{\text{max}}}{1+|{\text{x}}_{\text{d}}^{\text{″}}-{\text{x}}_{\text{T}}|\sin{\text{φ}}_{\text{nG}}}$$

(4) $${\text{K}}_{\text{S}0}=\frac{{\text{U}}_{\text{nQ}}}{{\text{U}}_{\text{nG}}\left(1+{\text{p}}_{\text{T}}\right)}\text{∗}\frac{1}{{\text{m}}_{\text{T}}}\text{∗}\left(1\pm {\text{p}}_{\text{T}}\right)\text{∗}\frac{{\text{c}}_{\text{max}}}{1+{\text{x}}_{\text{d}}^{\text{″}}\;\sin{\text{φ}}_{\text{nG}}}$$

The impedance factor of correction eq.s from eq. (1 -4) is derived by the equivalent voltage source supper position method comparison calculation results based on Thevenin's principle. Approximations that are applied for the derivations of the above expression are R ≪ X, $\sqrt{1+2{\text{x}}^{\text{″}}\;\sin\text{φ}+{\text{x}}^{\text{″}2}}$ $≅1+{\text{x}}^{\text{″}}\;\sin\text{φ}$ [7].

#### 3.1.1 Fault contribution of the main grid (MG)

Fault contribution of MG is the fault only without the integration of any type of power source and with a fault that happened to the system (with any type of fault). The fault happens at bus A and the magnitude of the fault is expressed as Eq. (5) [7].

**Figure 2:**
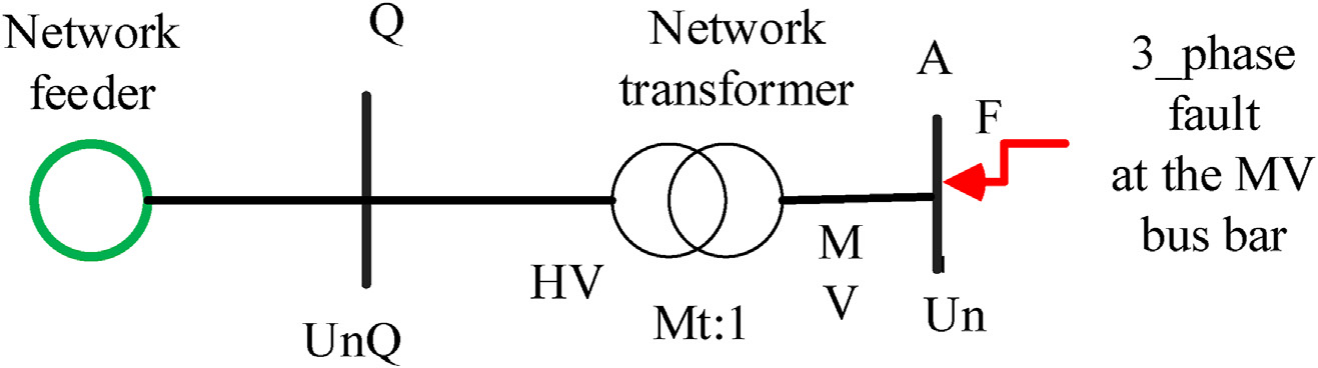
Fault contribution of the main grid.

Figure 2 is used for the calculation of the main grid fault level contribution which is shown in Eq. (5).

(5) $$I_{K}^{"}=\frac{c_{max}U_{n}}{\sqrt{3}\left(Z_{Q}+Z_{L}+Z_{R}+Z_{KT}\right)}$$

where $Z_{Q}$: main grid impedance at the connection point Q, $Z_{T}$: transformer impedance, $Z_{R}$: series reactor (R) (if any), $K_{T}$: correction factor, $Z_{L}$: line impedance.

**Figure 3:**
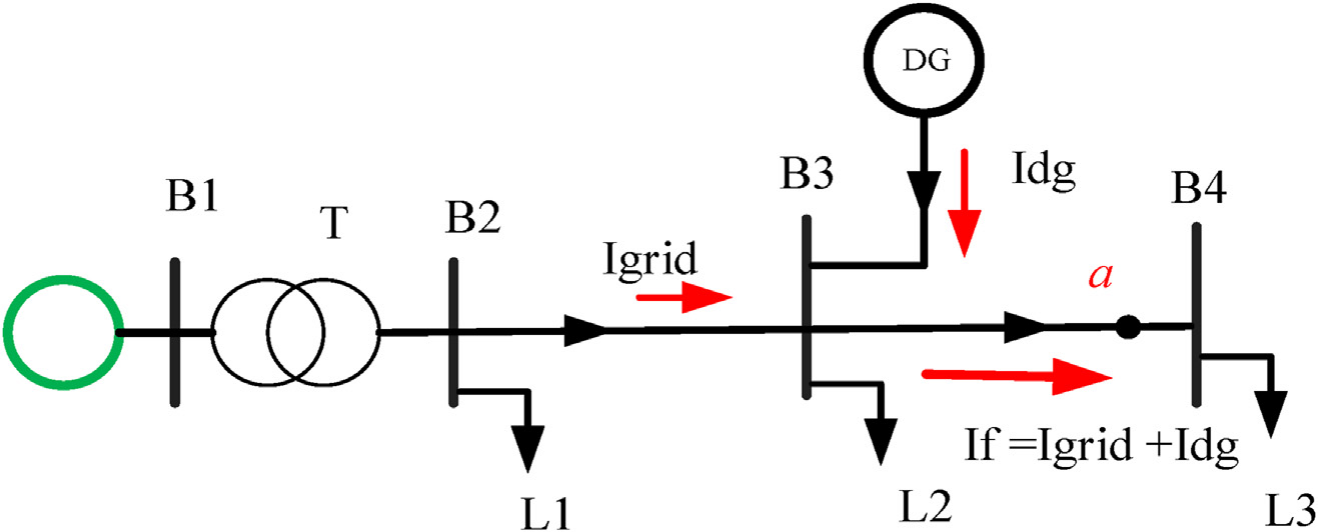
Fault contribution of DG.

Quantities that are calculated above are obtained by using Eq. (6).

$$Z_{Q}=\frac{cU_{n}}{\sqrt{3}I_{KQ}^{"}}$$

$$Z_{T}=\frac{u_{kr}}{100\%}*\frac{U_{rT}^{2}}{S_{rT}^{2}}$$

$$R_{T}=\frac{u_{Rr}}{100\%}*\frac{U_{rT}^{"2}}{S_{rT}^{2}}=\frac{P_{krT}}{3I_{rT}^{2}}$$

(6) $$K_{T}=0.95*\frac{c_{max}}{1+0.6x_{T}}*\frac{U_{rT}^{2}}{S_{rT}^{2}},\;X_{T}=\sqrt{Z_{T}^{2}-R_{T}^{2}}$$

where symbols $u_{kr}$, $P_{krT}$, and $I_{KQ}^{"}$ used in the calculations are fault voltage of the transformer, load loss at load current, and initial symmetrical fault current at the high voltage connection point Q respectively. After the integration of DG and fault happened at point a (fault location) near bus B4 the analysis becomes as Figure (3). The fault contribution of the grid and the DG is calculated as shown in Eq. (7) and the voltage measured by the relay is as Eq. (8).

(7) $$I_{K}^{"}=\frac{c_{max}U_{n}}{\sqrt{3}\left(Z_{G}+Z_{Q}+Z_{L}+Z_{R}+Z_{KT}\right)},\;{\text{I}}_{\text{f}}={\text{I}}_{\text{nw}}+{\text{I}}_{\text{DG}}$$

(8) $${\text{U}}_{\text{r}}={\text{I}}_{\text{nw}}\text{∗}{\text{Z}}_{23}+\left({\text{I}}_{\text{nw}}+{\text{I}}_{\text{DG}}\right)\text{∗}{\text{Z}}_{3\text{a}},\;{\text{Z}}_{\text{r}}=\frac{{\text{U}}_{\text{r}}}{{\text{I}}_{\text{nw}}}={\text{Z}}_{23}+{\text{Z}}_{3\text{a}}+\frac{{\text{I}}_{\text{dg}}}{{\text{I}}_{\text{nw}}}\text{∗}{\text{Z}}_{3\text{a}},$$

where: ${\text{Z}}_{\text{r}}$: apparent impedance, ${\text{U}}_{\text{r}}$: voltage measured by the relay, ${\text{ ​I}}_{\text{nw}}$: grid current, ${\text{Z}}_{23}$: line impedance from bus b_2_ to b_3_, ${\text{I}}_{\text{dg}}$: DG current contribution, ${\text{I}}_{\text{f}}$: fault current, ${\text{Z}}_{3\text{a}}$: impedance between z_3_ and fault location (point a).

#### 3.1.2 Reverse Power Flow and Voltage Profile

**Figure 4:**
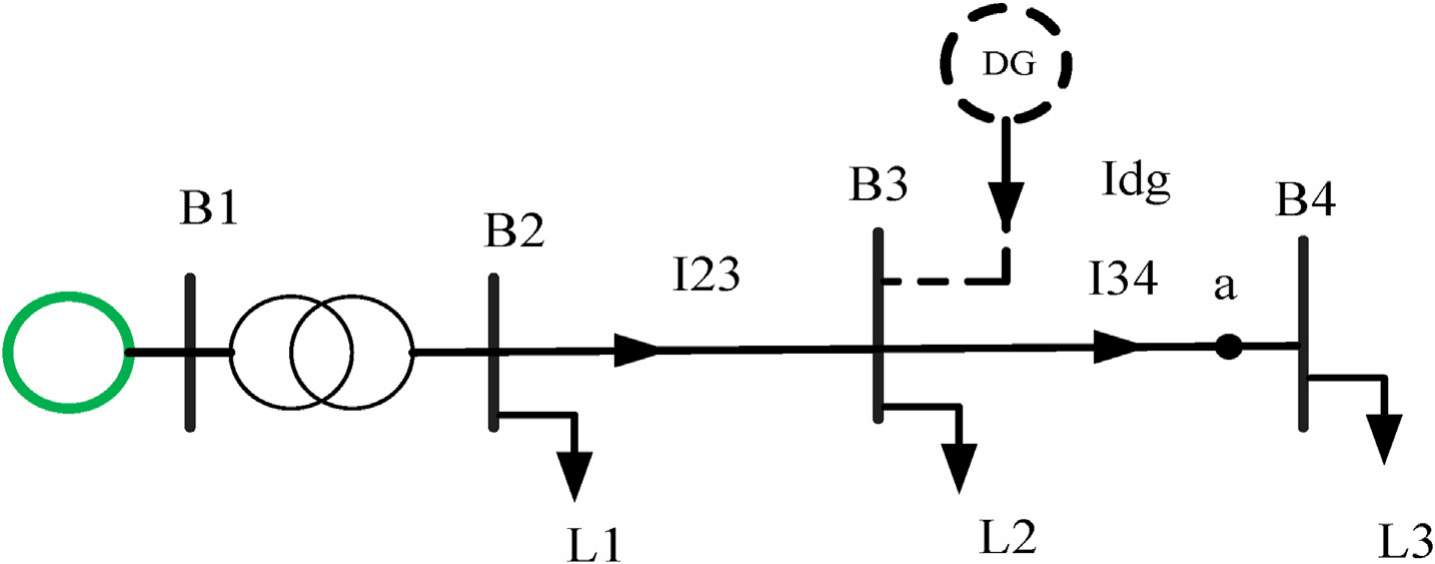
Reverse power flow and voltage profile.

Before the integration of DG, the DN has characteristics of radial nature. After the integration of DG, these characteristics are altered. Production of power greater than the consumer consumption or the fault happening in the system changes the direction of power. Protection of power system design must have to consider the reverse power flow if not it is problematic and it implies gradient reverse voltage along with the radial network. Integrating DG to the DN increases the voltage profile as shown in Eq. (9) below [8] and the voltage profile is calculated by using Figure 4.

(9) $$\Delta \text{U}\approx \frac{{\text{P}}_{\text{dg}}\text{∗}{\text{R}}_{\text{th}}+{\text{Q}}_{\text{dg}}\text{∗}{\text{X}}_{\text{th}}}{{\text{U}}_{\text{n}}}$$

where U_n_: nominal system voltage, R_th_ + j X_th:_ line impedance, and P_dg_ + j Q_dg_: DG power.

### 3.2 DG influence on protection scheme of distribution network

Integration of DG has influences on the protections of the DN schemes. These influences are protection blinding, untrue or false tripping, prevention or prohibition of automatic reclosing, reverse power flow, and lack of synchronization of automatic reclosing [6].

## 4 Power system protection

Making production, transmission, and distribution of electrical power to the demand as safe as possible from the effect of failure and preventing the power system from risk is called power system protection. The power system protection goals are public safety, equipment protection, and power system integrity [9]. The popularity of DG is increased due to the increment of demand for energy and its emphasis on clean energy. The power system has a nature of radially flowing which the power flows from the generation to the power demand. The integration of DG into the network alters the radial nature of the bidirectional flow of power. There are different problems that DG causes. These causes are protection device losing, feeders false trip, generators false trip, short circuit level increment and decrement and blinding of protection. Protection coordination depends on the capacity, type, and location of the DG. The criterion that the power system protection which are fundamentals of protection coordination must have satisfied the following five. These are reliability, selectivity, speed, simplicity, and economics [10].

### 4.1 Reverse power relay

It is a device used for the detection of reverse power of generator sets connected in parallel and supervision of active power to the power system. It also supervises the power direction for generators operating parallel with the main or another generator. At the time of failure of the prime mover of the generator/alternator, it operates as a motor and drives the prime mover. This leads to the loss of power and prime mover damage. When this type of operation is happened the RPR senses the reverse power direction switches off the alternator in case of this error. In addition to this, RPR can measure LL and LN voltage possible, time of tripping for supervision power and reverse power-adjustable. The comparison of conventional protection equipment and reverse power relay is discussed in detail in [11]. RPR is also commonly used in electrical power system synchronous generators detecting monitoring action at the time of failure of prime mover in the connection continuity of the excitation system [12]. Protecting reverse power flow by using RPR is effective and reliable for DG installation and RPR has the characteristics of protecting power system equipment from damage by reverse power flow with supplying continuous power to the demand [13].

#### 4.1.1 Modeling of reverse power relay

The element which has an important value for identifying the abnormal condition and modeling the relay is the reverse element of the relay. Power relays can measure the system current and voltage and the angle between the two angles θ. By using this information, the real power can be calculated as Eq. (10):

(10) P=V∗Icosθ

Real power flow is determined in forward and reverses direction in −90°<θ<90° and 90°<θ<270° respectively on under normal condition operations. The most important in identifying an abnormal is the reverse element of the relay.

#### 4.1.2 Reverse power level definition

A relay set is chosen for reverse power protection in a generator with its type [14] and its setting is as Eq. (11).

(11) $$\text{setting}=\frac{mottoring\;power\left(\%\right)*generating\;capacity\left(MVA\right)}{CT_{ratio}*VT_{ratio}}$$

#### 4.1.3 Trip time definition

Fake isolation under transient reversal of power is avoided by RPRs which can be built-in timers or external timers. Power reversal may be happened due to synchronization or disturbance of the transmission system. As the generating capacity increases the delay time should be decreased for the case of effective protection [13].

#### 4.1.4 Reverse power measuring principle

By multiplying the actual voltage and current values p(t)=v(t)∗i(t), the phase power can be calculated by the microprocessor. The instantaneous values of the calculated power are recorded and measured per cycle. The one cycle power can be calculated by using the Eq. (12).

(12) $$P=\frac{1}{T}\overset{2\pi }{\underset{0}{\int }}p\left(t\right)dt=\frac{1}{T}\overset{2\pi }{\underset{0}{\int }}\left(v\left(t\right)*i\left(t\right)\right)dt$$

The total power for the three-phase current is calculated by summing every single phase.

#### 4.1.5 Relay operating time

The time multiplier setting of the relay is the measure of the operating time of the relay and the user sets according to the requirements. The operating time of the relay can be changed by changing the plug setting multiplier (PSM) or time setting multiplier (T.S.M) or by changing simultaneously but the instantaneous time of the relay is unchangeable for the operating time. However, 20 ms–50 ms time delay was provided by the manufacturer generally [15]. PSM is calculated as Eq. (13).

(13) $$\text{PSM}=\frac{\text{Fault}\;\text{current}\;\text{in}\;\text{relay}\;\text{coil}}{\text{Pick}\;\text{up}\;\text{current}}=\frac{\text{Fault}\;\text{current}\;\text{in}\;\text{relay}\;\text{coil}}{\text{rated}\;\text{CT}\;\text{secondary}\;\text{current∗plug}\;\text{setting}}$$

For different relay characteristics, the operating time formula becomes Eq. (14):

$${\text{T}}_{\text{op}}=\frac{\text{K}}{\left(\text{P}.\text{S}.\text{M}-1\right)}T.M.S\;\left(long\;time\;inverse\right)$$

$${\text{T}}_{\text{op}}=\frac{\text{K}}{{\left(\text{P}.\text{S}.\text{M}\right)}^{2}-1)}(extremly\;inverse\;relay)$$

$${\text{T}}_{\text{op}}=\frac{K}{\left(\text{P}.\text{S}.\text{M}-1\right)}=T.M.S\;\left(very\;inverse\;relay\right)$$

(14) $${\text{T}}_{\text{op}}=\frac{\text{K}}{{\left(\text{P}.\text{S}.\text{M}\right)}^{0.02}-1)}T.M.S\;(standard\;inverse)$$

The coefficient (K) for different types of relays in international electro-technical commission standards are used to calculate the characteristics of operating time for different relays.

The coefficient (K) for different types of relays in international electro-technical Commission (IEC) standards which are used to calculate the characteristics of operating time for different relays are as below in Table 1).

**Table 1:** Coefficient for different types of relay.

	IEC	
	K	E
Normal inverse	0.14	0.02
Very inverse	13.5	1
Extremely inverse	80	2
Long time inverse	120	1

#### 4.1.6 Reverse power setting value calculation

For calculations of the setting value of the reverse power transformer, transformation ratio is used which is the way that the switching point has to be calculated. By assuming as the power is symmetrical the RPR measures the transformer secondary side of one phase power and the phase power of the generator must have to relate with the secondary side of the transformer. The essential data that can be used for the calculations of setting values of the reverse power are the rated apparent power of the generator, the rated power factor of the generator, RPR rated current, RPR rated voltage, current transformer transformation ratio, and potential transformer ratio [11].

#### 4.1.7 Behavior of reverse power relay

Characteristics of RPR are the indicators of the equipment to work with the correct connection of power system protection. The behavior or characteristics of the RPR covers a various system of connection behavior differences and expected improper operation of the connected system.

#### 4.1.8 Reverse power analysis flow chart for reverse power relay

**Figure 5:**
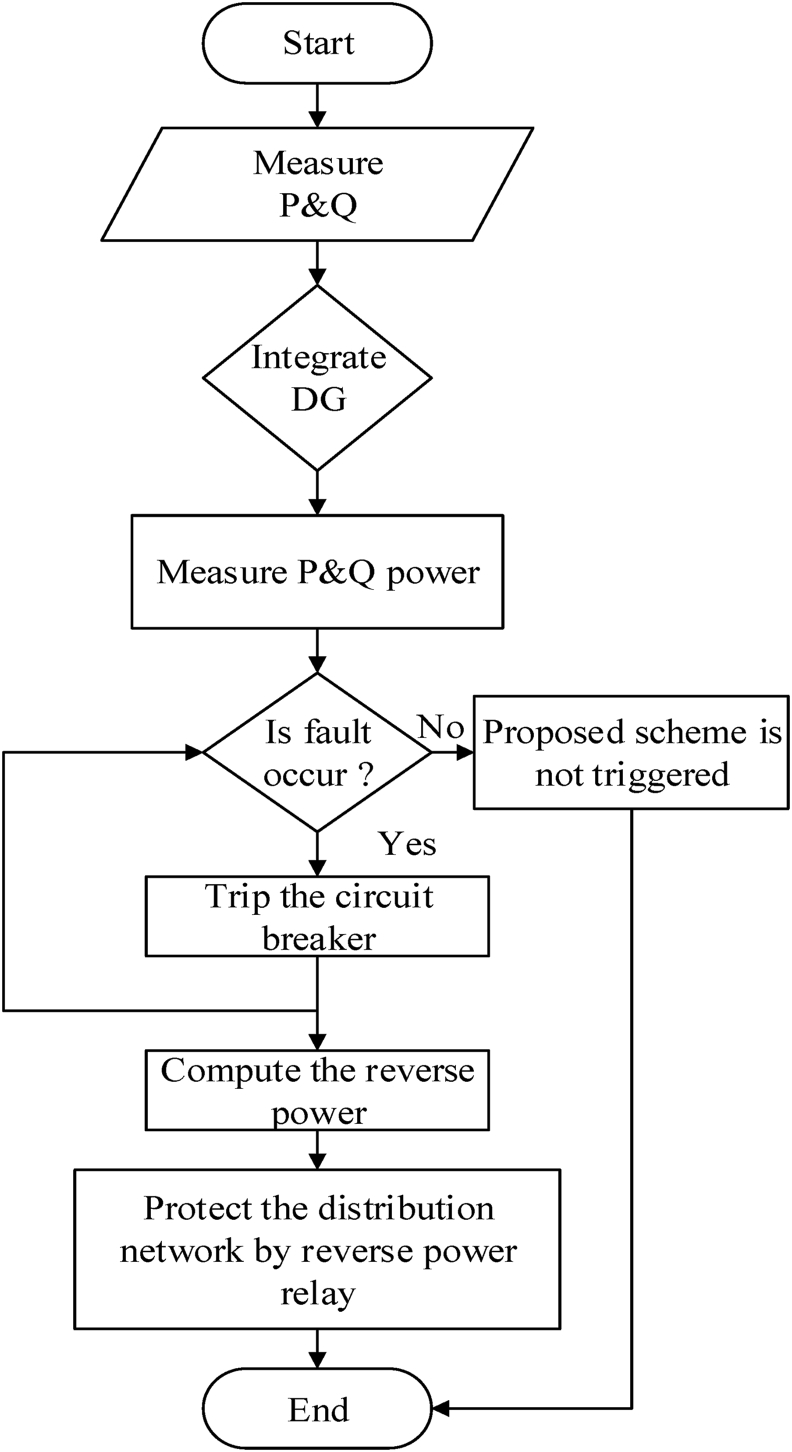
Flow chart analysis diagram.

Figure 5 shows the reverse power analysis flow chart for the reverse power relay.

### 4.2 Unidirectional fault current limiter

The FCL can be placed between the main grid and the DG. Since FCL has a bidirectional characteristic to limit the fault current, it can limit the fault current both from the main grid and the DG. But this degrades the reliability of the DG, main grid and DG overcurrent relays coordination in the case of a fault in the DG [16]. This drawback of FCL leads to placing UFCL between the main grid and the DG overcomes the influence of DG on the relays coordination and the time interval of current coordination is preserved among sorts of protective relays. And also placement of UFCL at the tie node makes the contribution of the main grid minimal to fault currents to DG and not vice versa [17].

#### 4.2.1 Principle of UFCL and structure

**Figure 6:**
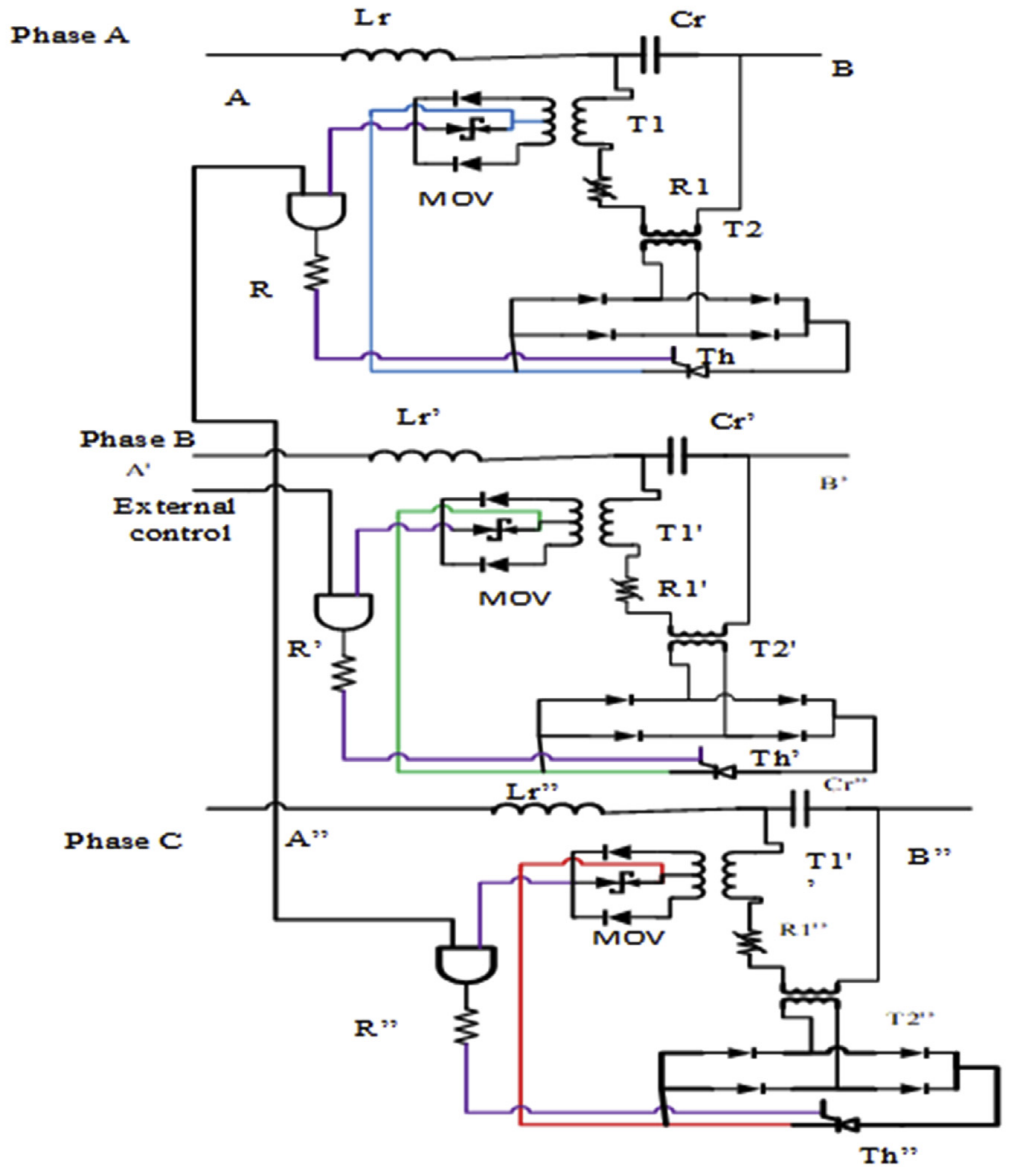
Proposed UFCL model.

**Figure 7:**
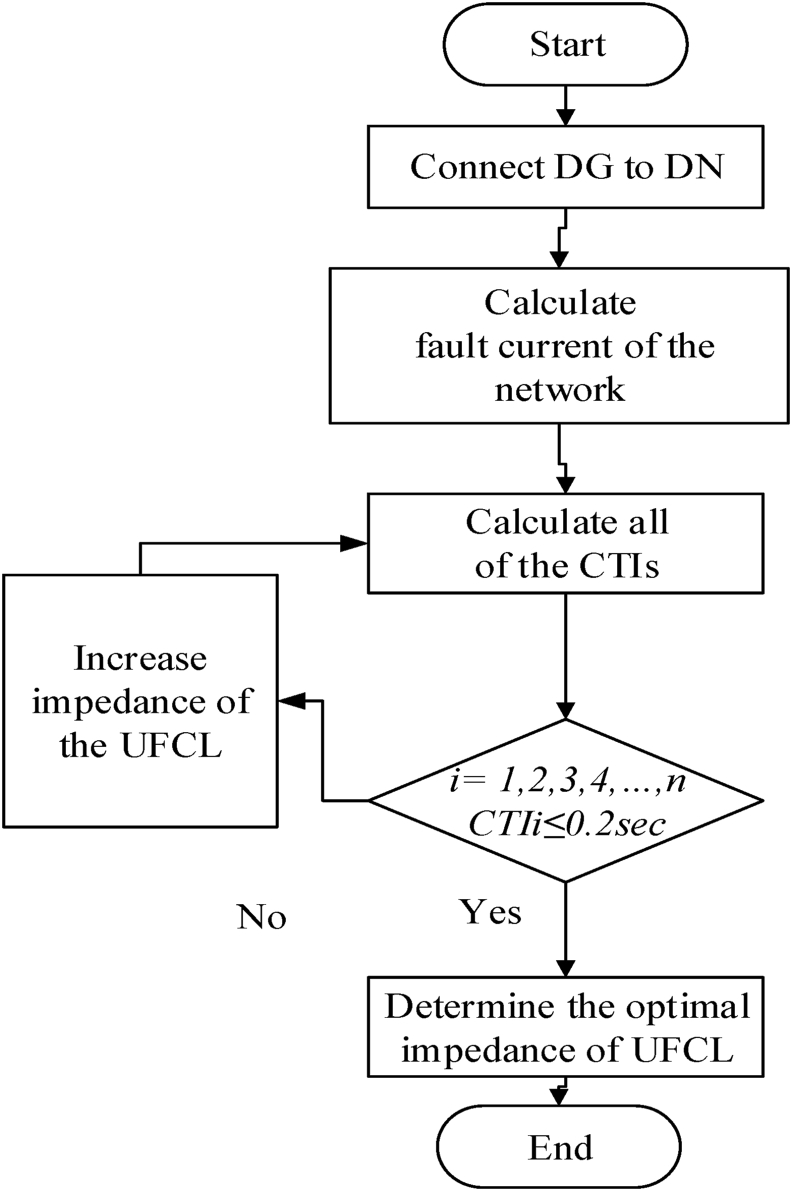
Impedance calculation flow.

This research proposes UFCL for the protection of the DG from the main grid fault condition where the model and optimal impedance determination flow chart of UFCL is as Figure 6 and Figure 7 respectively. In addition to this, research proposes UFCL with the principle of impedance values at the presence and absence of fault. The impedance value is low at the time of normal operation and conditions of fault at the DG. In another way, the impedance value increases at the conditions of fault at the main grid. UFCL is a device that is a series element and can prevent the fault from the main grid and protects the DG from fault. Based on the impedance value of UFCL there are two conditions in its principle [16]. These conditions are:

i. Low impedance (at normal and fault at DG conditions)
ii. High impedance (during fault at the main grid condition)

The above two conditions also follow another two basic conditions for the fulfillment of the protection capability of UFCL. The conditions are the series-parallel connections of the resonance circuit for the increment and decrement of impedance classified as low or high for the faulty and healthy conditions. The proposed UFCL is shown the Figure 6 below and it is the three-phase circuit topology of the proposed UFCL. The two conditions are:

a) Series circuit (Cr and Lr) at the normal condition

In this case transformer, T1 has a value of high inductance which is seen by Cr and it does not bother about the series resonant performed by (Cr – Lr). Since voltage drop is low across Cr and the transformer T2 secondary current is low, the metal oxide varistor (MOV) is active which cannot supply voltage to the gate of the thyristor (Th1) and the Th1 is OFF state. By the above conditions of the transformer T1, transformer T2, MOV, and Th1; the transformer T1 secondary winding is open effectively which orders the magnetizing inductance value of Cr.

b) Parallel condition (CR and Lr) at fault condition

Short-circuiting the secondary side of transformer T1 makes the resonant circuit parallel and the inductance value seen by Cr is reduced to a small leakage value. The voltage across Cr has increased whether the fault is the main grid or in the DG which is the activator of the increment for the primary current of T2. The MOV starts to operate due to the increment of the current of the transformer secondary winding of T2 and Th1 is activated to make the secondary of T1 short circuit. For this reason transformer, T1 inductance is going to a minimum to its leakage reactance which is seen by Cr and it makes a parallel resonant circuit. The insertion of current limiting impedance is done by UFCL. As Figure 3 shows there is an external command which is used to disable UFCL at the time of fault at DG.

#### 4.2.2 UFCL optimal impedance determination flow chart

This section includes the fault analysis in base case, location of DG, comparison of fault with and without UFCL installed, comparison of fault with and without reverse power relay installed and finally the conclusion. Figure 7 shows the flow chart to calculate the optimal impedance of UFCL.

## 5 Result and discussion

### 5.1 Base-case fault current level

The base case fault current calculation is shown which is calculated without the integration of DG to the system and considering all the 62 buses as fault location turn by turn. This helps the researcher to know the impact of DG in the DN protection to the fault level by distance of the fault location in the integration of DG. The maximum and minimum fault current level of 3_phase fault in KA is 0.860 and 0.613 respectively. The fault current level can mitigate only by the designed protective devices installed and the system does not need additional equipment's to protect fault from damaging the electrical devices.

**Figure 8:**
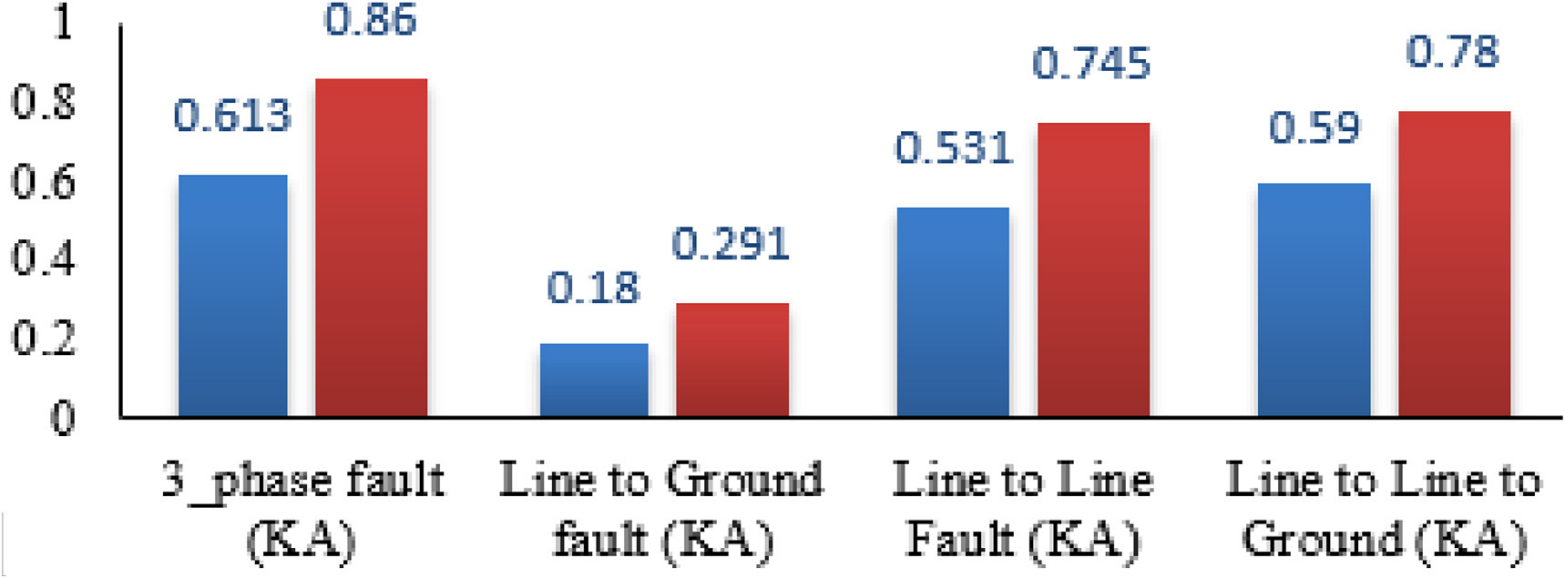
Base case Maximum and minimum fault current level.

The severity ranks of the faults starting from one to four are three phase (with 0.613KA minimum and 0.860KA maximum), double line to ground fault (with minimum 0.590KA and maximum 0.780KA), line to line fault (with minimum 0.531KA and maximum 0.745KA) and single line to ground (with minimum 0.180KA and maximum 0.291KA). This indicates that the theoretical and simulation result are correctly related and it shows a good result for the base case study. The protective device for the base case is designed based on the maximum fault that happens to the DN. The fault current level can mitigate only by the designed protective devices installed and the system does have not additional equipment to protect the fault from damaging the electrical devices. The fault current analysis considers three-phase, line to line, the line to ground, and double line to ground faults. The analysis is done by using ETAP 16.0 software and Figure 8 shows the maximum and minimum values of faults from any of the 62 buses. This is the base case that the paper going to show before the integrations of DGs where the DGs are selected as solar DG and wind DG in the case of this paper.

### 5.2 Locations of DG

**Figure 9:**
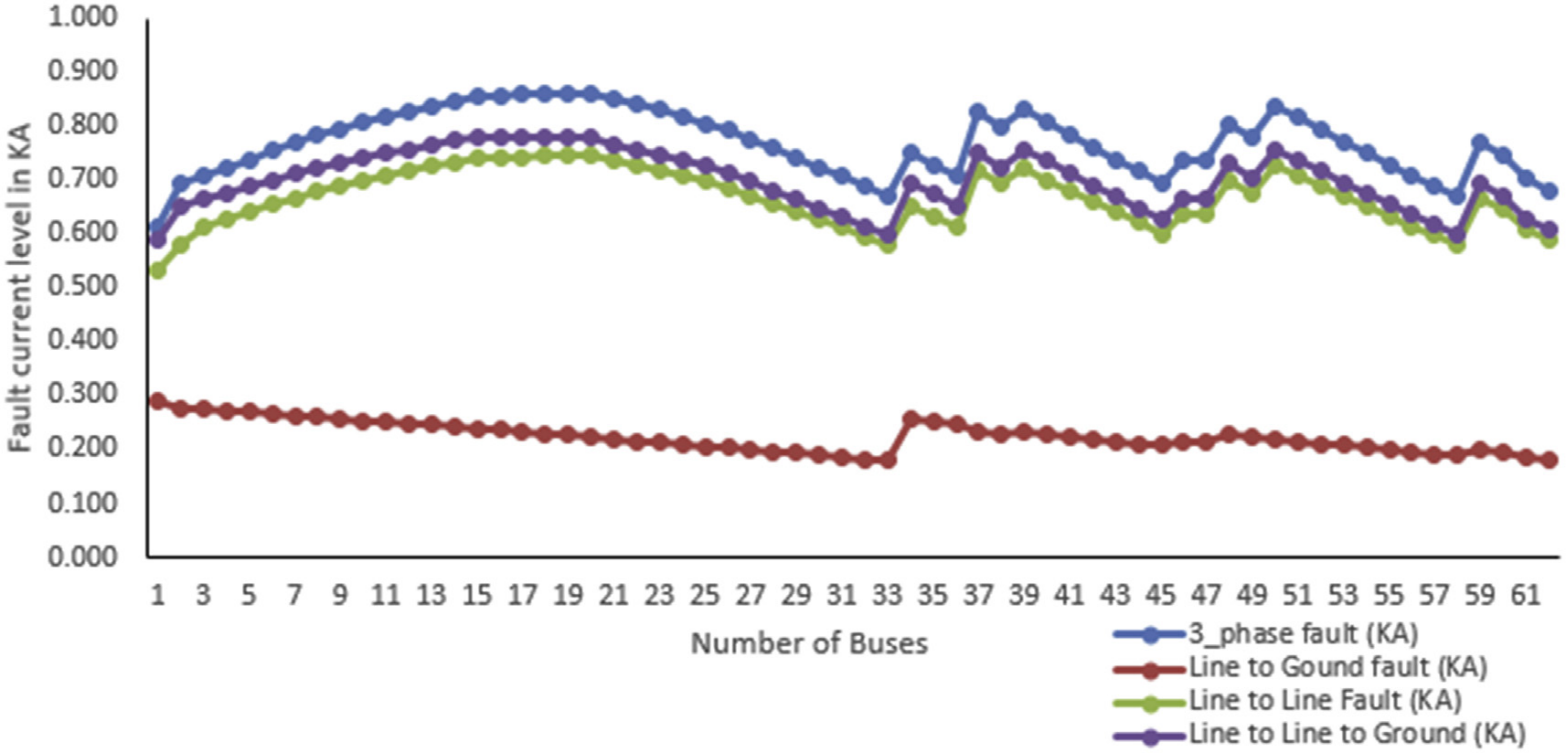
Base case fault current level analysis for the 62 buses DN.

**Figure 10:**
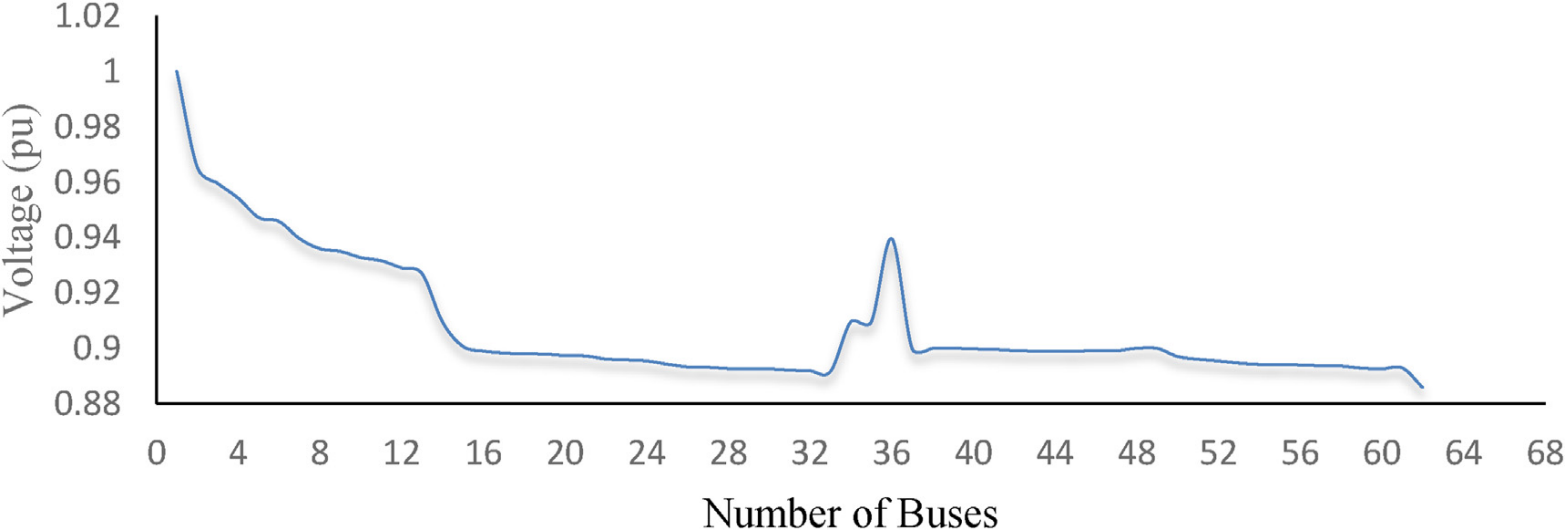
Base case voltage profile (PU) of the 62 bus DN.

**Figure 11:**
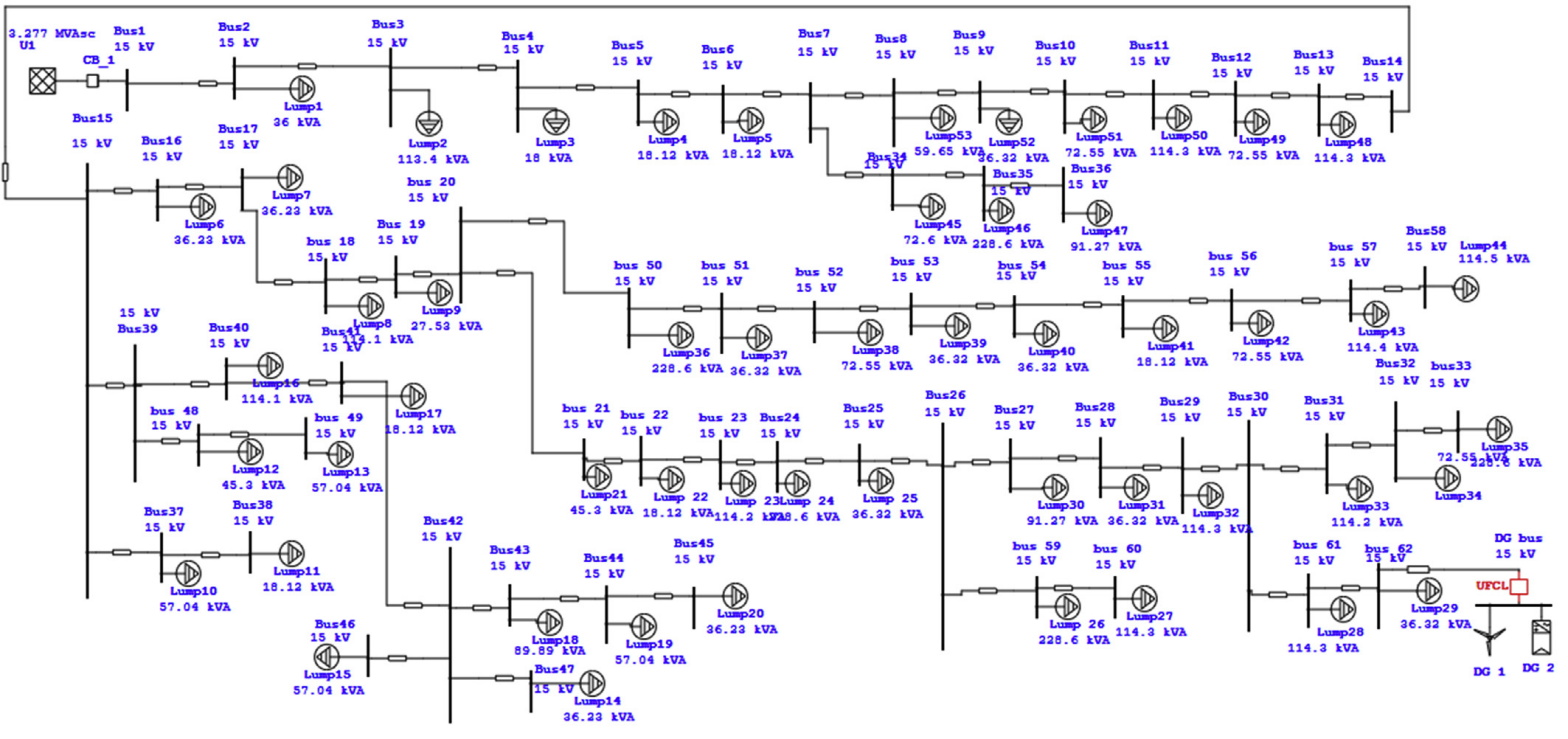
Comparison of fault with and without UFCL installed.

By using the loss sensitivity factor this paper identifies four weak buses. These buses are bus 31, bus 32, bus 33, and bus 62 as indicated in Figure 9 of the voltage profile (see Figures 10 and 11).

The selected buses are used for the placement of DG and analysis of fault level to know the adverse impact of DG. The adverse impact, which is discussed in this paper and has a great impact to increase fault current level to the DN is a type of DG (solar and wind), location of DG (4 selected weak buses these are bus 31, bus 32, bus 33 and bus 62), number of DG (two which are wind DG and solar DG located at a time and individually) and distance from the fault location. The voltage profile for the weakest bus is located at bus 62 with a value of 0.8859pu. The other weak buses are bus 31 with a voltage profile of 0.8922pu, bus 32 with a voltage profile of 0.8918pu, and bus 33 with a voltage profile of 0.8917pu. Table 2 shows the fault level of the system after the integration of solar and winds DG at the four weak buses which are bus 31, bus 32, bus 32, and bus 62 for knowing the contribution fault current of DG and the DS in each weak buses only. The fault location with a fixed DG placement has various magnitude of fault current levels. From the fault locations selected the weakest buses are listed shown in Table 2 below with the fault current level.

**Table 2:** Solar and wind DG and types of fault.

	3_phase (KA)				LG (KA)			
FL	Bus 31	Bus 32	Bus 33	Bus 62	Bus 31	Bus 32	Bus 33	Bus 62
31	1.236	1.224	1.212	1.178	0.933	0.886	0.844	0.789
32	1.179	1.217	1.205	1.125	0.859	0.926	0.879	0.735
33	1.125	1.160	1.198	1.075	0.795	0.977	0.918	0.687
62	1.123	1.113	1.103	1.211	0.763	0.731	0.703	0.923

	LL (KA)				DLG (KA)			
FL	Bus 31	Bus 32	Bus 33	Bus 62	Bus 31	Bus 32	Bus 33	Bus 62
31	1.080	1.069	1.058	1.028	1.152	1.130	1.111	1.077
32	1.029	1.063	1.052	0.981	1.097	1.137	1.116	1.030
33	0.982	1.013	1.046	0.937	1.046	1.082	1.123	0.986
62	0.980	0.970	0.961	1.058	1.037	1.021	1.008	1.133

An increasing number of DG have great support for the increment of fault current level to the system.

### 5.3 Comparison of fault with and without UFCL installed

**Figure 12:**
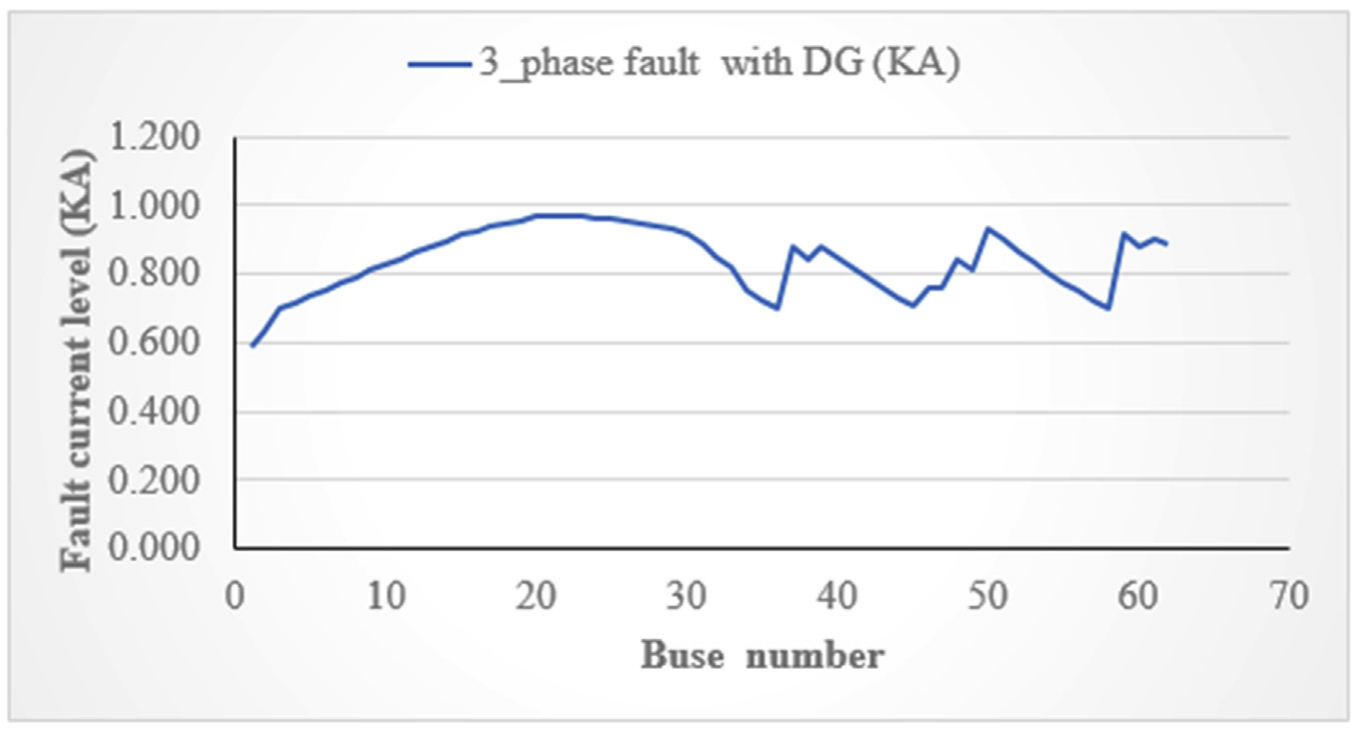
UFCL and DG installed DN.

The study selects only the two DGs connected at the time and the comparison is done on the weakest DG which is bus 62. All 62 buses are considered as fault locations to have a good discussion for the adverse impact of DG to their type and distance from the fault point and size of DG. The weakest bus (from the 62 buses) is bus number 62 which is identified by the loss sensitivity factor. The fault current level with the integration of UFCL and DG in DN is shown in Figure 12.

The fault current level at bus 62 before and after the integration of UFCL is as the table below (see Table 3).

**Table 3:** Fault current level before and after integration of UFCL with DG.

Fault type	Without UFCL	With UFCL	Improved (%)
Three-phase fault (KA)	1.211	0.886	26.84

### 5.4 Comparison of fault with and without reverse power relay installed

Reverse power means changing the direction of the flow of power which indicates changing generating characteristics of the generator to motoring characteristics. To prevent the reverse power RPR is proposed and its function is detecting the reverse power and giving a signal to the circuit breaker to be tripped. The relay sends a signal to the circuit breaker when the set minimum threshold or 180^0^ out of phase (which are current and voltage). The RPR reads and tries to monitor the fault current level and send a signal to the circuit breaker to be tripped at the settled time or the phase-out time in the amount of 180^0^. The duration of fault time controls the protection devices’ life and performance to protect fault from damages and injuries of personnel safety. Therefore, the integration of RPR is coordinated to the main circuit breaker to trip lesser than the time before the circuit breaker is tripped and minimized by 0.2 seconds.

## 6 Conclusion

This research work discusses the mitigation of fault current by using UFCL and RPR as mitigation techniques for the protection of Debre Markos DN (feeder 4) with its 62 bus in it. The mitigation of fault current is considering only the solar DG and wind DG. The fault analysis is done by using ETAP software. The base fault current at bus 62 for three-phase is 0.682KA and after the integration of solar and wind DG at a time for three-phase becomes 1.211KA. The fault current level after the integration of wind and solar DG is mitigated by UFCL is 0.886 KA for three-phase fault and it limits 0.325KA for 3Φ and the RPR is integrated at the point of common coupling of DG and DN. After the integration of RPR, the maximum level of reverse current before the integration of RPR is 1.211KA and it is tripped after 0.8 second and after the integration of RPR, the circuit breaker is tripped after 0.6 s.

The duration of fault time controls the protection devices’ life and performance to protect fault from damages and injuries of personnel safety. Therefore, the integration of RPR is coordinated to the main circuit breaker to trip lesser than the time before the circuit breaker is tripped and minimized by 0.2 seconds.

## Declarations

### Author contribution statement

Haymanot Takele: Conceived and designed the experiments; Performed the experiments; Analyzed and interpreted the data; Contributed reagents, materials, analysis tools or data; Wrote the paper.

### Funding statement

This research did not receive any specific grant from funding agencies in the public, commercial, or not-for-profit sectors.

### Data availability statement

Data included in article/supplementary material/referenced in article.

### Declaration of interest’s statement

The authors declare no conflict of interest.

### Additional information

No additional information is available for this paper.
